# Development of gastric mucosa-associated microbiota in autoimmune gastritis with neuroendocrine tumors

**DOI:** 10.1007/s00535-025-02298-w

**Published:** 2025-09-11

**Authors:** Koji Otani, Geicho Nakatsu, Kosuke Fujimoto, Daichi Miyaoka, Noriaki Sato, Yuji Nadatani, Yu Nishida, Hirotsugu Maruyama, Masaki Ominami, Shusei Fukunaga, Shuhei Hosomi, Fumio Tanaka, Seiya Imoto, Satoshi Uematsu, Toshio Watanabe, Yasuhiro Fujiwara

**Affiliations:** 1 Department of Gastroenterology, Osaka Metropolitan University Graduate School of Medicine, 10/F, 1-4-3 Asahimachi, Abeno-ku, Osaka, 545-8585 Japan; 2 Department of Immunology and Infectious Diseases, Harvard T.H. Chan School of Public Health, Room 904, Building 1, 665 Huntington Avenue, Boston, MA 02115 USA; 3 Department of Immunology and Genomics, Osaka Metropolitan University Graduate School of Medicine, 18/F, 1-4-3 Asahimachi, Abeno-ku, Osaka, 545-8585 Japan; 4 Division of Metagenome Medicine, Human Genome Center, The Institute of Medical Science, The University of Tokyo, 4-6-1 Shirokanedai, Minato-ku, Tokyo, 108-8639 Japan; 5 Division of Health Medical Intelligence, Human Genome Center, The Institute of Medical Science, The University of Tokyo, 4-6-1 Shirokanedai, Minato-ku, Tokyo, 108-8639 Japan; 6 Department of Premier Preventive Medicine, Osaka Metropolitan University Graduate School of Medicine, 12/F, 1-4-3 Asahimachi, Abeno-ku, Osaka, 545-8585 Japan

**Keywords:** Autoimmune gastritis, Neuroendocrine tumors, Gastric microbiota, Dysbiosis, Metabolomics

## Abstract

**Background:**

Autoimmune gastritis (AIG) is a chronic atrophic gastritis that affects the gastric corpus, leading to achlorhydria, hypergastrinemia, and a precursor of neuroendocrine tumors (NETs). This study aimed to elucidate the underlying mechanisms of gastric NET formation in AIG by analyzing gastric mucosa-associated microbiota and host tissue-derived metabolite profiles.

**Methods:**

A total of 19 patients diagnosed with AIG and 12 controls uninfected with *Helicobacter pylori* underwent gastric mucosal biopsies for microbiome analysis using next-generation sequencing with primers targeting the V3–V4 region of the 16S rRNA gene, and metabolome analysis using capillary electrophoresis time-of-flight mass spectrometry.

**Results:**

Microbiome analysis revealed significantly reduced α-diversity indices in patients with AIG when compared with the control group. β-Diversity analysis showed distinct microbial compositions among the control, NET-negative, and NET-positive groups. The NET-positive group exhibited a significantly higher abundance of Proteobacteria and Fusobacteriota, particularly *Haemophilus parainfluenzae*, *Fusobacterium periodonticum*, and *Fusobacterium nucleatum*, whereas Firmicutes, including *Streptococcus salivarius* and *Veillonella atypica*, were significantly decreased compared with the NET-negative group. Metabolome analysis revealed a shift away from glycolysis and tricarboxylic acid cycle activity toward alternative metabolic pathways in patients with AIG. Integrated analysis of gastric microbiota signatures (GMS) and tissue metabotypes demonstrated significant associations among GMS, tissue metabotypes, and NET diagnosis.

**Conclusions:**

These findings highlight marked shifts in gastric mucosa-associated microbiota profiles in patients with AIG who developed gastric NETs. Tissue-specific metabolic alterations may precede mucosal dysbiosis in patients with AIG and promote the development of a microenvironment implicated in NET formation.

**Supplementary Information:**

The online version contains supplementary material available at https://doi.org/10.1007/s00535-025-02298-w.

## Introduction

Autoimmune gastritis (AIG) is characterized by chronic atrophic gastritis in the gastric corpus, with immune-mediated destruction of gastric parietal cells through antigastric parietal cell antibodies, leading to achlorhydria and hypergastrinemia [1]. The Japanese Gastroenterological Endoscopy Society-Affiliated Study Group proposed diagnostic criteria for advanced-stage AIG, which include endoscopic or histological findings indicative of AIG and presence of antigastric parietal cells or anti-intrinsic factor antibodies. The Committee of AIG Research group identified several endoscopic features of AIG, such as remnants of oxyntic mucosa, sticky adherent dense mucus, and scattered minute whitish protrusions, in addition to a reverse atrophy pattern [2]. In recent years, the diagnostic criteria and endoscopic features of AIG have been gradually elucidated, leading to a surge in interest and attention toward this disease.

The diagnostic significance of AIG lies in the assessment of high-risk groups for the formation of gastric neuroendocrine tumors (NETs), one of the most challenging complications of AIG. The Rindi classification is widely used for the categorization of gastric NETs, and AIG is a precursor of type I NETs [3]. The incidence of gastric NETs in patients with AIG has been reported to range from 0.7 to 15.4% [4]. Hypergastrinemia has been reported to be associated with gastric NETs in AIG [5] and to stimulate the proliferation of enterochromaffin-like (ECL) cells in the gastric mucosa, thereby increasing the risk of developing gastric NETs [6]. However, the detailed pathophysiology underlying the formation of gastric NETs in patients with AIG remains poorly understood.

The stomach, with its highly acidic environment, has long been considered hostile to most bacteria, except for the urease-producing *Helicobacter pylori*, which is a primary risk factor for gastric cancer [7]. However, molecular analyses have revealed that various bacteria, including those from the phyla Proteobacteria, Firmicutes, Actinobacteriota, Bacteroidota, and Fusobacteriota, contribute to the composition of the human gastric microbiota [8]. Our previous research demonstrated that dysbiosis, defined as an alteration in the diversity or composition of gastric mucosa-associated microbiota persists even after *H. pylori* eradication therapy and underlies the development of metachronous gastric cancer [9]. Dysbiosis of gastric mucosa-associated microbiota is also observed in AIG, which is characterized by achlorhydria, and it has been reported that AIG is associated with a different microbial profile from that observed in *H. pylori*-induced atrophic gastritis [10]. The microbiota of the gastrointestinal (GI) tract, beyond the stomach, has been implicated in various autoimmune diseases, including inflammatory bowel disease [11, 12]. Furthermore, while there are studies that have investigated the association between GI microbiota and adenocarcinomas [13], very few studies have examined the relationship between GI microbiota and NETs. Furthermore, the relationship between NETs and metabolic pathways has been investigated through metabolome analysis of plasma from patients with NETs [14]. However, there are currently no reports on the alterations in metabolic pathways in patients with AIG.

Herein, we analyzed alterations in the gastric tissue microbiome and metabolic profiles and investigated their potential contributions to the pathogenesis of AIG and the formation of gastric NETs.

## Methods

### Study design

This was a single-center, observational study.

### Study population

A total of 19 AIG patients and 12 controls who are uninfected with *H. pylori* without AIG, were consecutively enrolled. Among the 19 AIG patients, seven patients who developed gastric NETs based on the histopathological examination were classified into the NET-positive group, while the remaining 12 patients who had never developed gastric NETs were classified into the NET-negative group.

### Eligibility criteria of the study population

Inclusion criteria were as follows: Among the patients aged ≥ 18 years who consented to participate at Osaka Metropolitan University Hospital between October 2020 and February 2023, those who were definitively diagnosed with AIG based on the diagnostic criteria for AIG described below were consecutively enrolled in the AIG group. In contrast, patients who were endoscopically assessed as uninfected with *H. pylori*, had no *H. pylori* eradication history, and were confirmed to be *H. pylori*-negative by serological test for anti-*H. pylori* antibody, histopathological examination, or stool *H. pylori* antigen test were matched to AIG patients by age (within a 10-year difference) and sex, and enrolled in the control group.

Exclusion criteria were as follows: Patients who either did not consent to participate or subsequently declined participation after being contacted were excluded.

### Diagnostic criteria for autoimmune gastritis

In this study, AIG was diagnosed based on the presence of antigastric parietal cell antibodies or anti-intrinsic factor antibodies, extensive atrophy of the gastric corpus observed during upper GI endoscopy, and achlorhydria confirmed through 24-h impedance-pH monitoring.

### Outcome measurement

The primary aim of this study was to investigate the development of gastric mucosa-associated microbiota and host tissue-derived metabolite profiles in AIG patients with and without gastric NETs and to elucidate the underlying mechanisms of gastric NET formation in AIG.

### Sample collection

Gastric mucosal biopsy samples were collected from all participants from four predefined sites during upper GI endoscopy: the greater curvature of the antrum (A1), lesser curvature of the angulus (A2), lesser curvature of the corpus (B1), and greater curvature of the corpus (B2). The samples were immediately frozen for microbiome and metabolome analyses.

### Medications

Patients discontinued acid-suppressing agents 2 weeks prior to undergoing 24-h impedance-pH monitoring for the diagnosis of AIG. After achlorhydria was confirmed and AIG was diagnosed, they were not prescribed any acid-suppressing agents and were not taking Such medications at the time of gastric mucosal biopsy for microbiome and metabolome analyses. Patients in the control group also discontinued acid-suppressing therapy 2 weeks prior to undergoing gastric mucosal biopsy. No patients receiving regular antibiotic therapy were included in this study.

### DNA extraction for microbiome analysis

Gastric mucosal biopsy samples were placed in a microcentrifuge tube containing 10 × Tris–EDTA (TE) buffer. Lysozyme solution (Lysozyme from chicken egg white; Merck KGaA, Darmstadt, Germany) was added, and the mixture was incubated at 37 °C for 1 h. Achromopeptidase (Achromopeptidase, Purified, Lytic Enzyme [TBL-1]; FUJIFILM Wako Pure Chemical Corp., Osaka, Japan) was then added, followed by incubation at 37 °C for 30 min. Proteinase K (Proteinase K from *Tritirachium album*; Merck) and 20% Sodium Dodecyl Sulfate solution (FUJIFILM Wako Pure Chemical Corp.) were Subsequently added, and the mixture was incubated at 55 °C for 1 h. Phenol–chloroform extraction was performed by adding UltraPure™ Phenol:Chloroform:Isoamyl Alcohol (25:24:1, v/v) (Thermo Fisher Scientific, Inc., MA, USA), followed by centrifugation. The aqueous phase was transferred to a new tube, and 3 M sodium acetate (FUJIFILM Wako Pure Chemical Corp.) and isopropanol were added. After centrifugation, the pellet was washed with 75% ethanol, centrifuged again, and air-dried. The pellet was resuspended in Ribonuclease (DNase free) Glycerol Solution (Nippon Gene Co., Ltd., Tokyo, Japan) in 1 × TE buffer, and the mixture was incubated at 37 °C for 30 min. DNA was further purified by adding 20% polyethylene glycol solution (50% w/v Polyethylene glycol 6000; Hampton Research Corp., CA, USA), followed by centrifugation. The resulting pellet was washed with 75% ethanol, centrifuged, air-dried, and finally resuspended in 0.5 × TE buffer.

### Microbiome analysis

The V3–V4 amplicon sequences of the 16S rRNA genes were prepared as described previously [15, 16]. Briefly, 16S rRNA gene fragments, including the V3 and V4 regions were amplified by polymerase chain reaction (PCR) (forward primer: ACACGACGCTCTTCCGATCTCCTACGGGNGGCWGCAG; reverse primer: GACGTGTGCTCTTCCGATCTGACTACHVGGGTATCTAATCC) for 20–30 cycles, and the PCR products were purified with Agencourt AMPure beads (Beckman Coulter). Next, for sequencing, index sequences were added by running a second PCR with NEBNext Multiplex Oligos for Illumina (Dual Index Primers Set1, New England Biolabs) for eight cycles, and the products were purified using Agencourt AMpure beads. For each sample, an equal amount of each DNA amplicon library was mixed and sequenced on the MiSeq instrument (Illumina) using a MiSeq v3 Reagent kit with 15% PhiX.

### Sample preparation for metabolome analysis

Gastric mucosal biopsy tissue samples were placed in a homogenization tube with 50% acetonitrile/Milli-Q water (v/v) containing internal standards and homogenized under cooling conditions twice at 1,500 rpm for 120 s using a tissue homogenizer. After centrifugation (2300×*g*, 4 °C, 5 min), the Supernatant was centrifugally filtered through a Millipore 5-kDa cutoff filter (UltrafreeMC-PLHCC). Following centrifugation (9100×*g*, 4 °C, 120 min), ultrafiltration was performed, and the filtrate was dried. The dried residue was then dissolved in Milli-Q water and used for capillary electrophoresis time-of-flight mass spectrometry (CE-TOFMS) analysis [17]. The measurement of cationic metabolites in cation mode was performed with an Agilent CE-TOFMS system (Agilent Technologies Inc., CA, USA), instrument No. 13, connected by a fused silica capillary (50 μm i.d. × 80 cm total length). The measurement of anionic metabolites in anion mode was conducted with an Agilent CE-TOFMS system (Agilent Technologies), instrument No. 7, connected by a fused silica capillary (50 μm i.d. × 80 cm total length).

### Metabolome analysis

Metabolome analysis was performed using the Basic Scan package from Human Metabolome Technologies (HMT) (HMT Inc., Yamagata, Japan). The peaks detected by CE-TOFMS were automatically extracted based on a signal/noise ratio ≥ 3 using the automatic integration software MasterHands™ ver. 2.19.0.2 [18] to obtain peak information including peak area values, mass/charge ratio (*m*/*z*), and migration time (MT). The acquired peak areas were converted into relative areas. Since these data included adduct ions, such as Na⁺ and K⁺, as well as fragment ions resulting from dehydration and ammonium loss, these mass-related ions were removed. The remaining peaks were annotated according to the HMT metabolite library based on their *m*/*z* and MT values.

### Visualization of gastric microbiota signatures and tissue metabotypes by non-negative matrix factorization

Non-negative matrix factorization was performed as previously described [19]. The optimal number of gastric microbiota signatures (GMS) (Supplementary Materials 1–3) and tissue metabotypes, and the regularization ratio were determined by aggregating results from 10 repetitions of 3-by-3 bicross validation [19]. Silva reference (v132) and Greengenes2 databases were used for the alignment and classification of 16S rRNA sequencing, respectively [20, 21]. Microbial taxa and metabolites with a false discovery rate (FDR) < 0.05 and an absolute effect size ≥ 1 were selected and data were visualized in a heatmap using the ggplot and ggalign R packages. Radial plots and alluvial diagram were constructed using volcano3D and ggalluvial R packages, respectively.

### Statistical analysis

Continuous variables were analyzed using the Wilcoxon rank-sum test for comparisons between two groups and the Kruskal–Wallis test for comparisons among three groups, followed by Dunn’s multiple comparison test for post hoc comparisons. Categorical variables were assessed using Pearson’s chi-square test or Fisher’s exact test, as appropriate. For microbiome analysis, sequencing data were processed using the bioinformatics pipelines Mothur (https://mothur.org/) [22] and QIIME2 (https://qiime2.org/) [23]. Differences in group centroids were assessed using permutational multivariate analysis of variance (PERMANOVA) implemented in the vegan R package. For metabolome analysis, Welch’s *t* test and simple linear regression was performed for the comparative analysis between two groups. Statistical analyses were performed using R software (Version 4.3.1, The R Foundation for Statistical Computing, Vienna, Austria). Statistical significance was defined as *p* < 0.05. *p* values were adjusted according to the Benjamini–Hochberg method.

## Results

### Baseline characteristics of patients

There were no statistically significant differences in age and sex between the control, NET-negative, and NET-positive groups. The rates of antiparietal cell antibody positivity and hypergastrinemia (≥ 3000 pg/mL) were significantly higher, and the pepsinogen I/II ratio was significantly lower in both the NET-negative and NET-positive groups with AIG compared with the control group. When comparing the NET-negative and NET-positive groups, hypergastrinemia (≥ 3000 pg/mL) was more frequently observed in the NET-positive group than in the NET-negative group (85.7% vs. 50.0%, respectively), although the difference was not statistically significant (Table 1).

**Table 1:** Baseline characteristics of patients

Variables	CL	N−	N+	*p*-Value			
				Overall	CL vs. N−	CL vs. N+	N− vs. N+
Number of patients	12	12	7				
Age (median [IQR])	65.5 (55.5, 73.0)	63.0 (52.5, 72.3)	66.0 (53.0, 70.5)	0.946	0.772	0.899	0.799
Sex				0.989	1.000	1.000	1.000
Male	2 (16.7%)	2 (16.7%)	1 (14.3%)				
Female	10 (83.3%)	10 (83.3%)	6 (85.7%)				
Alcohol intake (≥ 5 days a week)	2 (16.7%)	3 (25.0%)	0 (0.0%)	0.365	1.000	0.497	0.263
Smoking habit (current smoker)	4 (33.3%)	2 (16.7%)	1 (14.3%)	0.520	0.640	0.603	1.000
BMI (kg/m^2^) (median [IQR])	24.8 (20.2, 29.1)	21.2 (19.5, 23.2)	22.2 (21.4, 22.5)	0.367	0.184	0.447	0.499
Anti-parietal cell antibody (+)	4 (33.3%)	12 (100.0%)	7 (100.0%)	< 0.001	0.001	0.013	1.000
Anti-intrinsic factor antibody (+)	0 (0.0%)	6 (50.0%)	2 (28.6%)	0.025	0.014	0.137	0.633
Pepsinogen I (ng/mL) (median [IQR])	161.0 (106.2, 196.5)	4.6 (3.0, 5.2)	5.7 (4.6, 10.7)	< 0.001	< 0.001	0.001	0.128
Pepsinogen II (ng/mL) (median [IQR])	23.3 (16.5, 28.2)	7.4 (5.9, 12.6)	9.6 (8.0, 11.0)	0.002	0.001	0.010	0.353
Pepsinogen I/II (median [IQR])	6.3 (5.4, 7.2)	0.5 (0.4, 0.7)	0.6 (0.6, 1.2)	< 0.001	< 0.001	< 0.001	0.201
Gastrin ≥ 3000 (pg/mL)	0 (0.0%)	6 (50.0%)	6 (85.7%)	0.001	0.014	< 0.001	0.173
Iron deficiency anemia	2 (16.7%)	0 (0.0%)	2 (28.6%)	0.177	0.478	0.603	0.123
Vitamin B12 deficiency anemia	0 (0.0%)	5 (41.7%)	2 (28.6%)	0.046	0.037	0.123	0.656
Anti-TPO antibody (+)	1 (8.3%)	6 (50.0%)	5 (71.4%)	0.014	0.069	0.010	0.633
Hypothyroidism	1 (8.3%)	1 (8.3%)	3 (42.9%)	0.092	1.000	0.117	0.117
Hypertension	1 (8.3%)	5 (41.7%)	3 (42.9%)	0.130	0.155	0.117	1.000
Dyslipidemia	3 (25.0%)	4 (33.3%)	3 (42.9%)	0.721	1.000	0.617	1.000
Diabetes mellitus	3 (25.0%)	2 (16.7%)	3 (42.9%)	0.451	1.000	0.617	0.305
*H. pylori* eradication history	0 (0.0%)	4 (33.3%)	2 (28.6%)	0.092	0.093	0.123	1.000
Anti-*H. pylori* antibody (U/mL, median [IQR])	0.0 (0.0, 2.0)	0.0 (0.0, 3.0)	0.0 (0.0, 1.5)	0.993	1.000	0.909	0.918
Gastric cancer history	0 (0.0%)	3 (25.0%)	1 (14.3%)	0.187	0.217	0.368	1.000

CL, control group; N−, neuroendocrine-negative group; N+, neuroendocrine-positive group; IQR, interquartile range; BMI, body mass index; TPO, thyroid peroxidase; *H. pylori*, *Helicobacter pylori*

### Upper gastrointestinal endoscopic and histopathological findings of the background gastric mucosa

The characteristic upper GI endoscopic findings associated with AIG include reverse atrophy, remnants of oxyntic mucosa, pseudopolyps, sticky adherent dense mucus, and scattered minute whitish protrusions. The prevalence of reverse atrophy, remnants of oxyntic mucosa, pseudopolyps, sticky adherent dense mucus, and ECL cell hyperplasia were significantly higher in both the NET-negative and NET-positive groups with AIG compared with the control group. When comparing the NET-negative and NET-positive groups, remnants of oxyntic mucosa and pseudopolyps were more frequently observed in the NET-positive group than in the NET-negative group (85.7% vs. 50.0% and 100.0% vs. 58.3%, respectively); however, no significant differences were observed in the overall frequency of these endoscopic findings. Evaluation of the histopathological findings of gastric mucosal biopsy specimens using the Updated Sydney system showed that the NET-negative group exhibited a significantly higher degree of intestinal metaplasia at the greater curvature of the corpus compared with the NET-positive group (*p* = 0.019). The positivity rate for ECL cell hyperplasia was higher in the NET-positive group than that in the NET-negative group (71.4% vs. 50.0%), although this difference was not statistically significant (Supplementary Material 4).

### Characteristics of neuroendocrine tumor lesions occurring in patients with autoimmune gastritis

There were 12 NETs, with a median size of 5.5 mm (interquartile range 4.5–6.0). Half of the NETs were located in the upper corpus. NETs appeared on endoscopy as Subepithelial tumors originating from the deep mucosal layer. According to the World Health Organization Classification, all NETs were well-differentiated NET grade 1 (Supplementary Material 5).

### Alterations in α-diversity of gastric mucosa-associated microbiota at the level of 16S rRNA gene operational taxonomic units

**Fig. 1:**
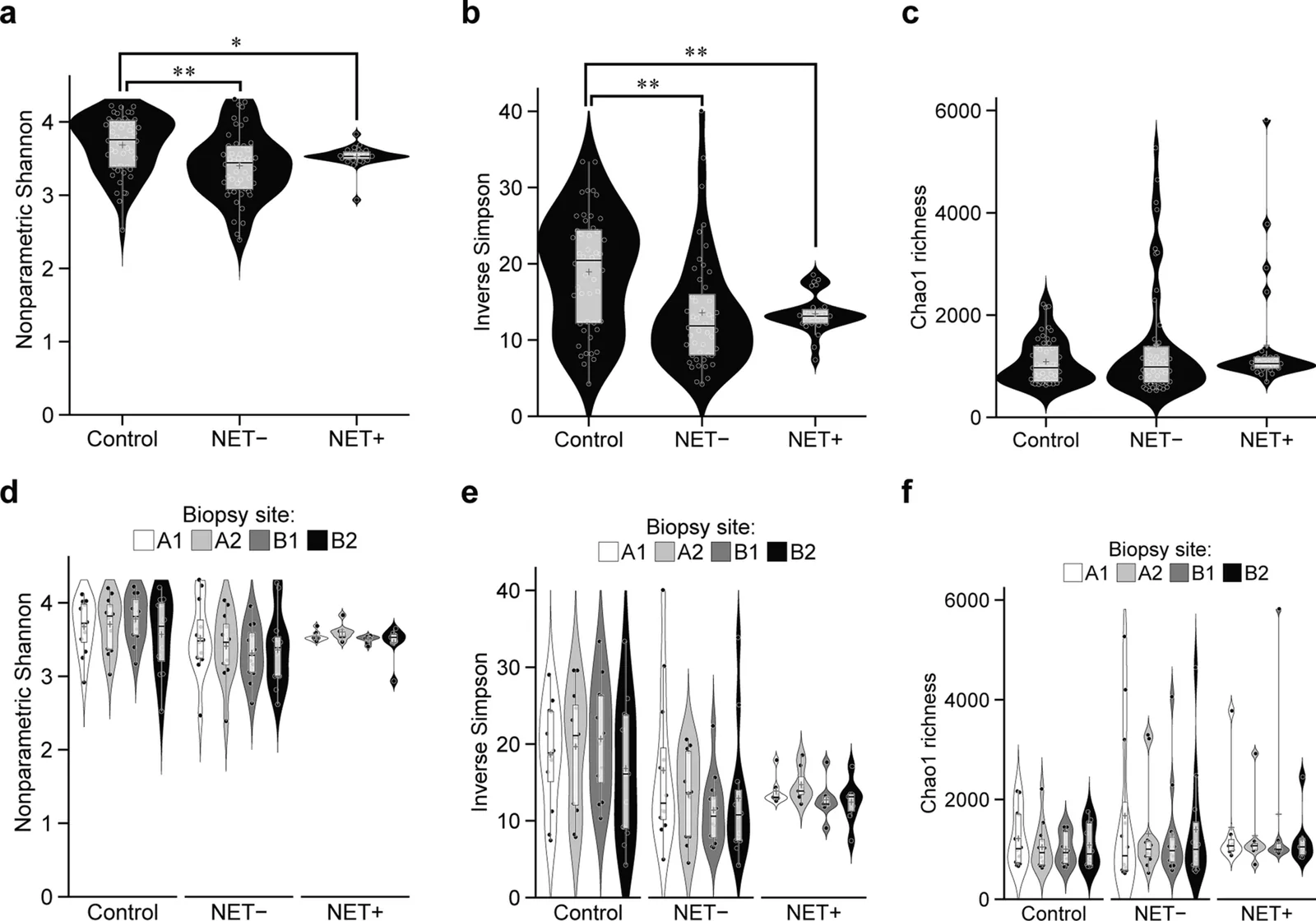
Alterations in α-diversity of gastric mucosa-associated microbiota at the level of 16S rRNA gene operational taxonomic units. **a** Nonparametric Shannon index, **b** inverse Simpson index, **c** Chao1 richness, **d** nonparametric Shannon index by biopsy site, **e** inverse Simpson index by biopsy site, **f** Chao1 richness by biopsy site. NET−, neuroendocrine tumor (NET)-negative group; NET+, NET-positive group; A1, Greater curvature of antrum; A2, Lesser curvature of angulus; B1, Lesser curvature of corpus; B2, Greater curvature of corpus; *p < 0.05; **p < 0.01

Among the α-diversity metrics, the nonparametric Shannon index was significantly lower in both the NET-negative and NET-positive groups with AIG compared with the control group (control vs. NET-negative group, *p* = 0.002, FDR = 0.022; control vs. NET-positive group, *p* = 0.012, FDR = 0.094) (Fig. 1a). Similarly, the Inverse Simpson index was significantly reduced in both the NET-negative and NET-positive groups with AIG compared with the control group (control vs. NET-negative group, *p* < 0.001, FDR = 0.007; control vs. NET-positive group, *p* = 0.003, FDR = 0.037) (Fig. 1b). The α-diversity metrics of nonparametric Shannon index, Inverse Simpson index, and Chao1 richness revealed that the NET-positive group exhibited a more compact distribution with lower variability (Fig. 1a–c). There were no significant differences in α-diversity metrics across the biopsy sampling sites within the same group (Fig. 1d–f).

### Alterations in β-diversity of gastric mucosa-associated microbiota at the level of 16S rRNA gene operational taxonomic units

**Fig. 2:**
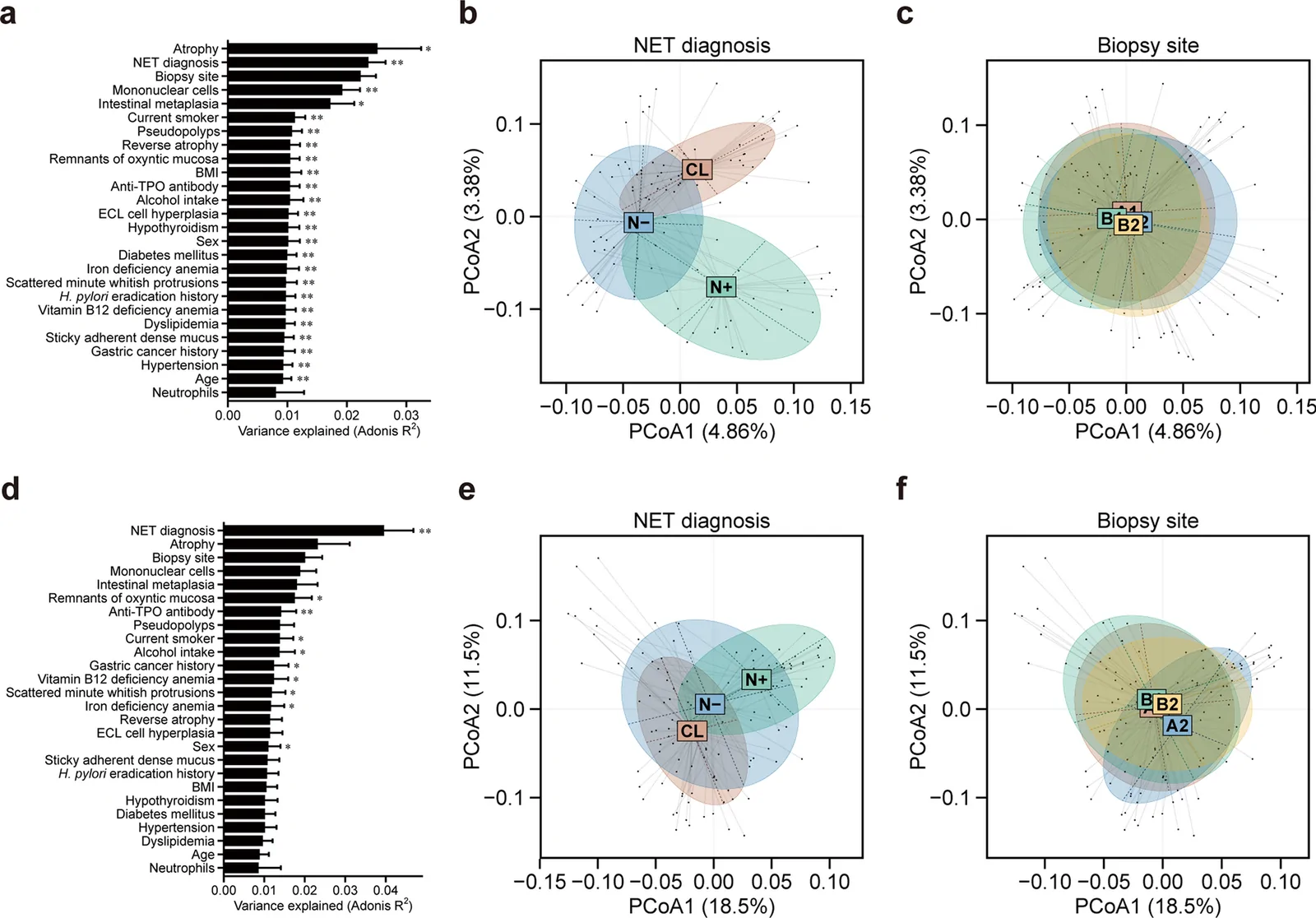
Alterations in β-diversity of gastric mucosa-associated microbiota at the level of 16S rRNA gene operational taxonomic units. **a** Permutational multivariate analysis of variance (PERMANOVA) based on unweighted UniFrac distance to assess the impact of factors on microbial community variation, **b** Principal coordinate analysis (PCoA) plot based on unweighted UniFrac distance by neuroendocrine tumor (NET) diagnosis, **c** PCoA plot based on unweighted UniFrac distance by biopsy site, **d** PERMANOVA based on weighted UniFrac distance to assess the impact of factors on microbial community variation, **e** PCoA plot based on weighted UniFrac distance by NET diagnosis, **f** PCoA plot based on weighted UniFrac distance by biopsy site. BMI, body mass index; TPO, thyroid peroxidase; ECL, enterochromaffin-like; *H. pylori*, *Helicobacter pylori*; CL, control group; N−, NET-negative group; N+, NET-positive group; A1, Greater curvature of antrum; A2, Lesser curvature of angulus; B1, Lesser curvature of corpus; B2, Greater curvature of corpus; *p < 0.05; **p < 0.01

In β-diversity analysis, PERMANOVA based on unweighted UniFrac distance revealed that NET diagnosis was a major determinant of microbial community variation among background factors (*R*^2^ = 0.023, *p* = 0.001, FDR = 0.005) (Fig. 2a). The principal coordinate analysis (PCoA) plot further demonstrated a significant separation in microbial community composition among the control, NET-negative, and NET-positive groups (Fig. 2b). In contrast, no significant differences were observed across the biopsy sampling sites within the same group (*R*^2^ = 0.022, *p* = 0.875, FDR = 0.875) (Fig. 2c). Similarly, PERMANOVA based on the weighted UniFrac distance showed that NET diagnosis accounted for the greatest variance in β-diversity among the background factors examined (*R*^2^ = 0.039, *p* = 0.001, FDR = 0.005) (Fig. 2d). The corresponding PCoA plot also revealed a significant separation in microbial community composition among the control, NET-negative, and NET-positive groups (Fig. 2e). Similar to the unweighted UniFrac analysis, no significant differences were detected across biopsy sampling sites within the same group (*R*^2^ = 0.020, *p* = 0.697, FDR = 0.714) (Fig. 2f).

### Comparative relative abundance analysis of changes in gastric mucosa-associated microbial taxa

Taxonomic abundance analysis showed that the NET-positive group exhibited significantly higher proportions of Proteobacteria (25.0% vs. 17.7%, *p* = 0.001) and Fusobacteriota (6.1% vs. 2.9%, *p* < 0.001) and a lower proportion of Firmicutes (38.8% vs. 49.2%, *p* < 0.001) compared with the NET-negative group at the phylum level. Among the phyla Proteobacteria, Fusobacteriota, and Firmicutes, the NET-positive group demonstrated significantly higher proportions of *Neisseria* (16.06% vs. 6.93%, *p* = 0.007) and *Fusobacterium* (4.10% vs. 1.46%, *p* < 0.001) and a lower proportion of *Veillonella* (4.31% vs. 6.61%, *p* = 0.002) and *Lactobacillus* (0.56% vs. 2.39%, *p* = 0.026) at the genus level. Among the major bacterial species with high relative abundances, the NET-positive group exhibited a significant increase in *Haemophilus parainfluenzae* (2.32% vs. 2.07%, *p* = 0.011) within the phylum Proteobacteria, as well as *Fusobacterium periodonticum* (3.01% vs. 0.82%, *p* < 0.001) and *Fusobacterium nucleatum* (0.25% vs. 0.14%, *p* = 0.002) within the phylum Fusobacteriota, compared with the NET-negative group. In contrast, *Streptococcus salivarius* (2.64% vs. 7.86%, *p* < 0.001) and *Veillonella atypica* (1.56% vs. 2.53%, *p* = 0.003) within the phylum Firmicutes were significantly decreased. Graphs illustrating the comparative abundance analysis of gastric mucosa-associated microbial taxa are presented in Supplementary Material 6. The relative abundance data and changes of gastric mucosa-associated microbiota (top 50 species after removal of unassigned organisms) are provided in Table 2.

**Table 2:** Comparative relative abundance analysis of changes in gastric mucosa-associated microbial taxa

Bacteria	Relative abundance (%)			*p*-Value			
	CL	N−	N+	Overall	CL vs. N−	CL vs. N+	N− vs. N+
Firmicutes							
*Streptococcus salivarius*	6.12	7.86	2.64	< 0.001	> 0.999	0.002	< 0.001
*Veillonella atypica*	2.98	2.53	1.56	< 0.001	> 0.999	< 0.001	0.003
*Lactobacillus gasseri*	0.32	1.29	0.07	0.004	0.643	0.003	0.074
*Streptococcus anginosus*	0.35	0.34	0.46	0.142	0.147	> 0.999	0.944
*Veillonella parvula*	0.14	0.51	0.02	0.034	0.562	0.438	0.029
*Lactobacillus murinus*	0.13	0.41	0.00	0.002	> 0.999	0.002	0.022
*Lactobacillus reuteri*	0.29	0.24	0.00	0.248	> 0.999	0.689	0.292
*Eubacterium sulci*	0.18	0.26	0.42	< 0.001	> 0.999	< 0.001	0.002
*Megasphaera micronuciformis*	0.45	0.42	0.44	0.928	> 0.999	> 0.999	> 0.999
*Dialister invisus*	0.28	0.02	0.00	< 0.001	0.068	< 0.001	0.129
*Lactobacillus fermentum*	0.06	0.17	0.23	0.253	0.297	> 0.999	> 0.999
*Streptococcus gordonii*	0.19	0.11	0.00	< 0.001	> 0.999	< 0.001	0.001
*Lactobacillus salivarius*	0.03	0.12	0.22	0.096	> 0.999	0.119	0.207
*Streptococcus constellatus*	0.15	0.10	0.00	< 0.001	0.111	< 0.001	0.010
*Filifactor alocis*	0.06	0.07	0.14	< 0.001	0.282	< 0.001	< 0.001
Proteobacteria							
*Haemophilus parainfluenzae*	1.35	2.07	2.32	0.004	> 0.999	0.006	0.011
*Acinetobacter guillouiae*	2.04	0.68	0.04	0.052	0.187	0.081	> 0.999
*Haemophilus influenzae*	0.91	0.51	0.22	0.177	> 0.999	0.195	0.541
*Brevundimonas diminuta*	0.56	0.13	0.01	0.203	0.485	0.328	> 0.999
*Haemophilus parahaemolyticus*	0.54	0.89	1.16	< 0.001	> 0.999	< 0.001	0.002
*Haemophilus haemolyticus*	0.13	0.14	0.46	< 0.001	> 0.999	< 0.001	< 0.001
*Acinetobacter baumannii*	0.00	0.29	0.03	0.289	> 0.999	0.356	> 0.999
*Comamonas testosterone*	0.29	0.02	0.01	0.082	0.195	0.159	> 0.999
*Bacteroidota*							
*Prevotella melaninogenica*	1.66	3.07	2.21	0.020	0.025	0.152	> 0.999
*Prevotella histicola*	2.53	1.54	0.68	0.003	> 0.999	0.003	0.009
*Porphyromonas pasteri*	0.32	0.66	2.28	< 0.001	> 0.999	< 0.001	< 0.001
*Prevotella jejuni*	1.05	1.56	0.75	0.761	> 0.999	> 0.999	> 0.999
*Prevotella salivae*	0.54	0.76	0.52	0.740	> 0.999	> 0.999	> 0.999
*Alloprevotella tannerae*	0.73	0.57	1.94	< 0.001	0.138	< 0.001	< 0.001
*Porphyromonas gingivalis*	0.50	0.39	0.89	< 0.001	0.065	0.008	< 0.001
*Prevotella pallens*	0.58	0.67	0.45	0.830	> 0.999	> 0.999	> 0.999
*Prevotella veroralis*	0.24	0.21	0.11	0.107	> 0.999	0.166	0.172
*Prevotella nigrescens*	0.32	0.13	0.13	0.458	0.669	> 0.999	> 0.999
*Prevotella intermedia*	0.25	0.09	0.52	< 0.001	0.556	< 0.001	< 0.001
*Prevotella nanceiensis*	0.31	0.28	0.89	< 0.001	> 0.999	< 0.001	< 0.001
*Prevotella baroniae*	0.12	0.10	0.20	< 0.001	> 0.999	0.002	< 0.001
*Prevotella aurantiaca*	0.04	0.18	0.36	< 0.001	> 0.999	< 0.001	< 0.001
*Porphyromonas endodontalis*	0.21	0.12	0.49	< 0.001	0.203	0.001	< 0.001
*Bacteroides cellulosilyticus*	0.25	0.03	0.00	0.290	> 0.999	0.433	0.503
*Prevotella oris*	0.15	0.10	0.03	0.005	> 0.999	0.007	0.016
*Prevotella denticola*	0.14	0.14	0.09	0.564	> 0.999	> 0.999	0.864
Fusobacteriota							
*Fusobacterium periodonticum*	0.54	0.82	3.01	< 0.001	> 0.999	< 0.001	< 0.001
*Fusobacterium nucleatum*	0.22	0.14	0.25	0.002	> 0.999	0.015	0.002
Actinobacteriota							
*Rothia_mucilaginosa*	1.10	0.51	1.47	< 0.001	0.304	0.001	< 0.001
*Schaalia odontolytica*	1.65	1.31	1.69	< 0.001	0.013	> 0.999	0.002
*Actinomyces graevenitzii*	0.77	0.69	0.45	0.320	> 0.999	0.397	> 0.999
*Corynebacterium argentoratense*	0.12	0.05	0.66	< 0.001	0.029	< 0.001	< 0.001
*Rothia aeria*	0.10	0.06	0.10	< 0.001	> 0.999	< 0.001	< 0.001
*Bifidobacterium longum*	0.23	0.06	0.01	0.016	0.878	0.012	0.146
Campilobacterota							
*Campylobacter concisus*	0.21	0.29	0.20	0.420	0.978	0.675	> 0.999

CL, control group; N−, neuroendocrine-negative group; N+, neuroendocrine-positive group

### Pathway-level changes in host tissue-derived metabolites

Metabolome analysis using CE-TOFMS detected 378 peaks (221 cationic and 157 anionic) based on the *m*/*z* and MT values registered in the HMT metabolite library. Metabolites with significant differences (*p* < 0.05 and ratio > 1.5-fold or < 0.67-fold) in the comparative analysis between the control and NET-negative groups along with the corresponding metabolic pathways are shown in Table 3. Metabolites related to glucose metabolism (glycolysis), tricarboxylic acid (TCA) metabolism, and coenzyme metabolism were decreased, whereas those associated with glucose metabolism (modification), glutamate metabolism, choline metabolism, tryptophan metabolism, and nucleic acid metabolism were increased in the NET-negative group of patients with AIG compared with the control group.

**Table 3:** Pathway-level changes in host tissue-derived metabolites

Metabolites and the metabolic pathways	CL vs. N−		CL vs. N+		N− vs. N+	
	Ratio (N-/CL)	*p*-Value	Ratio (N+/CL)	*p*-value	Ratio (N+/N−)	*p*-Value
Glucose metabolism						
Glycolysis						
Glucose 6-phosphate	0.60	< 0.001	0.63	0.001	1.05	0.719
Fructose 1,6-diphosphate	0.63	0.003	0.79	0.404	1.25	0.518
Hexosamine pathway (modification)						
N-Acetylglucosamine 6-phosphate	1.54	0.027	1.47	0.046	0.95	0.776
N-Acetylneuraminic acid	2.49	0.005	1.53	0.005	0.61	0.046
CMP-N-acetylneuraminate	1.95	< 0.001	1.28	0.069	0.66	0.011
Energy storage through TCA metabolism						
ATP	0.51	0.006	0.62	0.065	1.21	0.609
ADP	0.57	< 0.001	0.70	0.004	1.22	0.230
NAD^+^	0.37	< 0.001	0.50	< 0.001	1.33	0.136
NADP^+^	0.33	< 0.001	0.40	< 0.001	1.22	0.284
CoA	0.57	0.010	0.36	< 0.001	0.62	0.092
Energy conversion through TCA metabolism						
Carnitine	0.71	< 0.001	0.66	0.001	0.93	0.485
2-Aminoadipic acid	0.40	0.003	0.45	0.007	1.13	0.605
Saccharopine	0.16	< 0.001	0.21	0.001	1.30	0.099
Glutamate metabolism and the urea cycle						
Citrulline	2.15	0.011	1.28	0.133	0.60	0.049
GABA	5.44	< 0.001	2.45	0.008	0.45	0.004
Guanidoacetic acid	2.93	< 0.001	1.85	0.024	0.63	0.037
5-Oxoproline	2.61	0.010	1.78	0.230	0.68	0.300
N-Acetylputrescine	2.45	0.001	1.61	0.034	0.66	0.040
Imidazole-4-acetic acid	2.16	0.005	1.82	0.043	0.84	0.448
Asp	1.87	< 0.001	1.49	0.002	0.80	0.070
Gly	1.59	0.001	1.28	0.066	0.81	0.056
Choline metabolism and the methionine cycle						
Betaine aldehyde	3.77	0.006	1.46	0.042	0.39	0.016
Betaine	2.14	0.004	1.58	0.100	0.74	0.203
*O*-Phosphoserine	2.18	0.018	2.43	0.046	1.12	0.712
Ser	1.66	0.013	1.29	0.037	0.78	0.149
Met	1.45	0.088	0.92	0.463	0.64	0.046
Tryptophan Metabolism						
Kynurenine	2.10	0.001	1.72	0.006	0.82	0.233
Quinolinic acid	2.66	0.008	2.11	0.079	0.79	0.351
Nicotinamide	1.73	0.001	1.39	0.018	0.80	0.052
Nucleic acid metabolism–Purine metabolism–Adenosine/guanosine						
IMP	1.99	0.010	1.14	0.583	0.58	0.027
Adenylosuccinic acid	2.14	0.006	1.35	0.236	0.63	0.067
Adenosine	2.42	< 0.001	2.27	< 0.001	0.94	0.642
Guanosine	2.29	0.020	1.68	0.082	0.73	0.284
Nucleic acid metabolism–Pyrimidine metabolism						
Uracil	2.82	0.038	1.51	0.437	0.54	0.191
Uridine	2.38	0.040	1.48	0.368	0.62	0.245
Cytidine	1.91	0.043	1.15	0.593	0.60	0.115
CMP	1.77	< 0.001	1.37	0.055	0.77	0.076
Coenzyme metabolism						
Vitamin B2 metabolism						
FAD	0.62	< 0.001	0.55	< 0.001	0.90	0.191
Vitamin B5 metabolism						
Pantothenic acid	0.63	0.046	0.65	0.091	1.02	0.948
Vitamin B6 metabolism						
Pyridoxamine 5′-phosphate	0.43	< 0.001	0.40	< 0.001	0.93	0.443

CL, control group; N−, neuroendocrine tumor-negative group; N+, neuroendocrine tumor-positive group; CMP, cytidine monophosphate; TCA, tricarboxylic acid; ATP, adenosine triphosphate; ADP, adenosine diphosphate; NAD^+^, nicotinamide adenine dinucleotide; NADP^+^, nicotinamide adenine dinucleotide phosphate; CoA, coenzyme A; GABA, γ-aminobutyric acid; Asp, aspartic acid; Gly, glycine; Ser, serine; Met, methionine; IMP, inosine monophosphate; FAD, flavin adenine dinucleotide

### Identification of gastric microbiota signatures and tissue metabotypes by non-negative matrix factorization

**Fig. 3:**
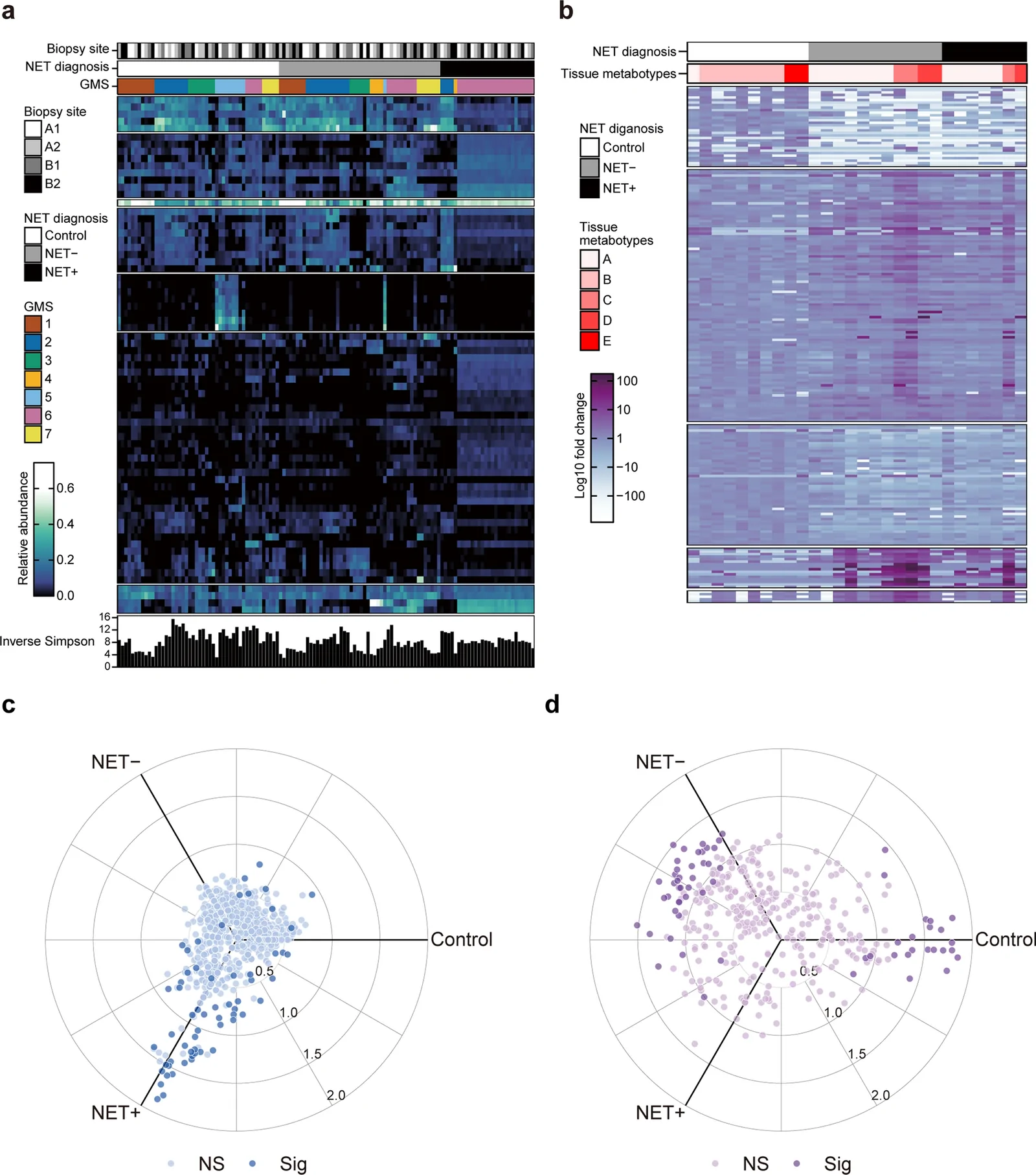
Identification of gastric microbiota signatures and tissue metabotypes by non-negative matrix factorization. **a** Heatmap visualization of the relative abundance of gastric mucosa-associated microbiota. **b** Heatmap visualization of the relative abundance (relative area under the peak) of host tissue-derived metabolites. Rows are microbial taxa or metabolite features, sorted by k-means clustering; columns represent individual microbiome samples or metabolic profiles, sorted by nested hierarchical ward D2 clustering. **c** Radial plots of shifts in the relative abundance of operational taxonomic units. **d** Radial plots of shifts in the relative abundance (relative area under the peak) of host tissue-derived metabolites. Axis values represent *z*-scores. NET, neuroendocrine tumor; GMS, gastric microbiota signatures; A1, Greater curvature of antrum; A2, Lesser curvature of angulus; B1, Lesser curvature of corpus; B2, Greater curvature of corpus; NET−, NET-negative group; NET+, NET-positive group; NS, not significant; Sig, significant

Heatmap visualization of the relative abundance of gastric mucosa-associated microbiota revealed a distinct compositional pattern in the NET-positive group compared with the control and NET-negative groups, with GMS6 being predominant in the NET-positive group (Fig. 3a). Host tissue-derived metabolite profiles demonstrated that the NET-negative and NET-positive groups in patients with AIG shared a comparable metabolite composition, whereas the metabolite composition shifted between the control group and patients with AIG patients; tissue metabotype B was specific to the control group, whereas tissue metabotype A was predominantly associated with patients with AIG (Fig. 3b). Radial plot analysis illustrated a shift in the relative abundance of specific microbial taxa toward the NET-positive group compared with the NET-negative and control groups (Fig. 3c). There were systematic differences in the relative abundance of host tissue-derived metabolites between patients with AIG and the control group (Fig. 3d).

### Integrated analysis of gastric microbiota signatures and tissue metabotypes

**Fig. 4:**
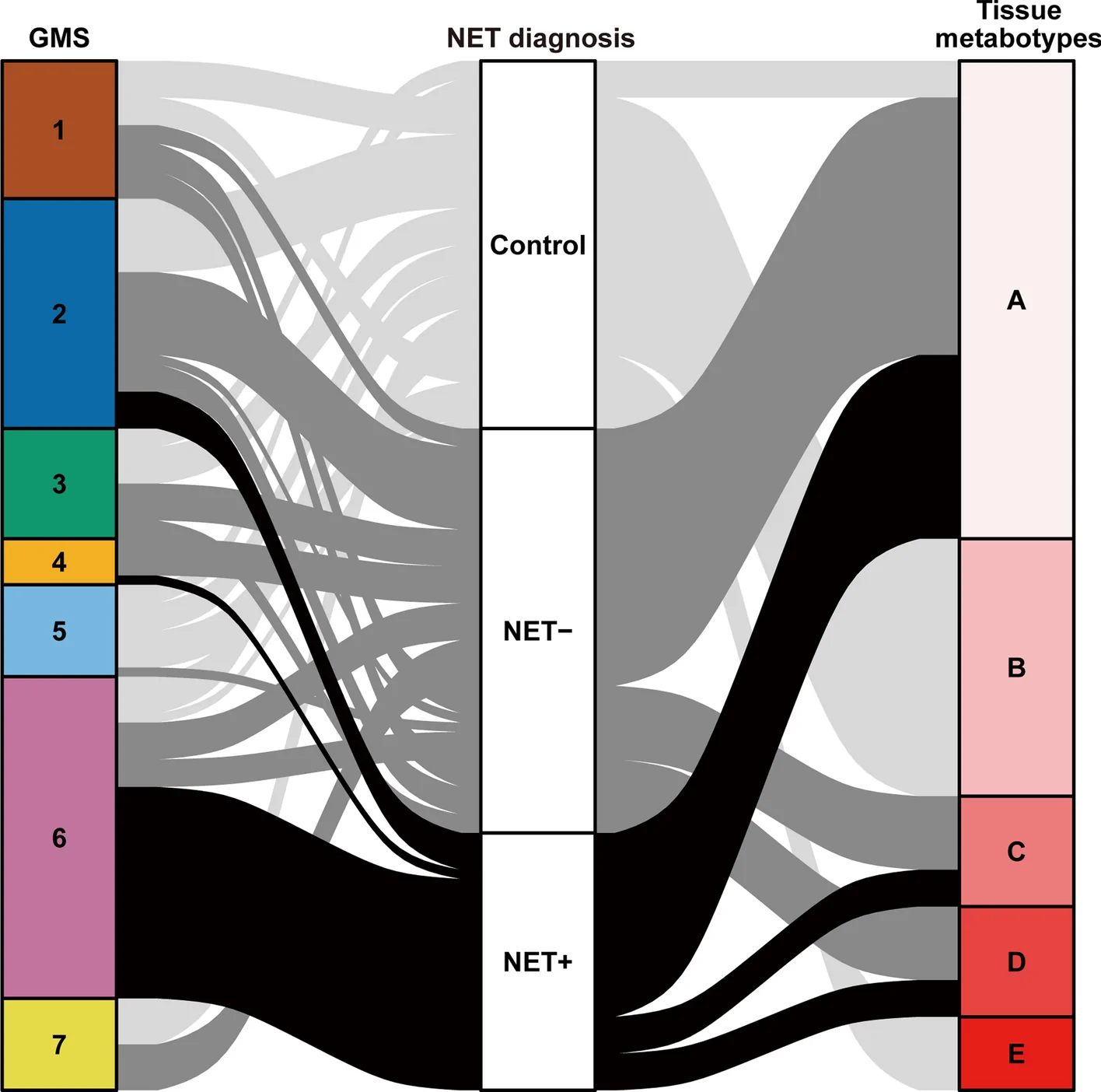
Integrated analysis of gastric microbiota signatures and tissue metabotypes. Alluvial diagram summarizing the relative frequency relationships among gastric microbiota signature, NET diagnosis, and tissue metabotypes. Grey, dark grey and black lines correspond to the control, NET-negative, and NET-positive groups, respectively. GMS, gastric microbiota signatures; NET, neuroendocrine tumor; NET−, NET-negative group; NET+, NET-positive group

An alluvial diagram generated from the integrated analysis of GMS and tissue metabotypes revealed significant associations among GMS, NET diagnosis, and tissue metabotypes (GMS vs. NET diagnosis, *p* = 1 × 10^–6^; NET diagnosis vs. tissue metabotypes, *p* = 1 × 10^–6^; GMS vs. tissue metabotypes, *p* = 4.7 × 10^–5^). In particular, prominent flow connections were observed between GMS6, the NET-positive group, and tissue metabotype A, as well as between the NET-negative group and tissue metabotype A (Fig. 4).

## Discussion

In this study, alterations in gastric mucosa-associated microbiota profiles were observed in patients with AIG who developed gastric NETs. This enhances our understanding of AIG pathophysiology from a previously unexplored perspective. Among the diversity of the microbiota, the α-diversity metrics, specifically the nonparametric Shannon index and Inverse Simpson index, were significantly reduced in gastric mucosa-associated microbiota of AIG patients. This finding is consistent with a previous report showing that Pielou’s evenness, species richness, and the Simpson index were significantly higher in the microbiota of normal stomachs compared with those of AIG patients [10]. This reduction indicates a less diverse microbiota in patients with AIG, which is consistent with the previous studies linking reduced microbial diversity to disease states, including GI malignancies [24–26]. Furthermore, the more compact distribution with lower variability observed in the NET-positive group suggests a more uniform microbiota composition, which may contribute to NET formation. The β-diversity analysis using unweighted and weighted UniFrac distances revealed that AIG and NET status were associated with distinct microbial compositions.

A heatmap of the bacterial community revealed a distinct microbiota profile in the NET-positive group, characterized by increased proportions of Proteobacteria and Fusobacteriota. Proteobacteria are Gram-negative bacteria that include a wide variety of pathogenic species responsible for human diseases [27]. Previous studies have reported an association between *Haemophilus parainfluenzae* and Crohn’s disease [28], as well as non-small cell lung cancer [29]. Fusobacteriota are anaerobic, gram-negative bacteria that constitute integral components of the oral and GI microbiomes. *Fusobacterium periodonticum* and *Fusobacterium nucleatum* are known to promote inflammation and cause pathogenicity. *Fusobacterium periodonticum* is associated with esophageal cancer [30] and oral cancer [31], and *Fusobacterium nucleatum* has been implicated in the development of ulcerative colitis [32] and the progression of colorectal cancer [33, 34] and oral cancer [35]. On the other hand, a concomitant decrease in Firmicutes was observed in the NET-positive group. Firmicutes are Gram-positive bacteria that include many beneficial species essential for maintaining human health. *Streptococcus salivarius* has been reported to exhibit anti-inflammatory properties [36], with salivaricins produced by this bacterium inhibiting the growth of oral pathogens [37]. Additionally, *Veillonella atypica* has been reported to improve treadmill runtime and reduce inflammatory cytokine levels in mice [38], while the probiotic *Veillonella atypica* has been observed to maintain exercise performance in human [39].

These alterations in gastric mucosa-associated microbiota suggest that microbial dysbiosis contributes to the formation of gastric NETs in patients with AIG. In the present study, hypergastrinemia and ECL cell hyperplasia were more frequently observed in the NET-positive group, although these differences were not statistically significant. These factors may have potentially contributed to alterations in the proportions of specific bacterial populations.

Gastric NETs associated with AIG typically arise in the fundic gland region, particularly in the gastric corpus and fundus [40], which is consistent with the areas of gastric mucosal atrophy observed in AIG. In this study, 91.7% of the NET lesions were located in the gastric corpus or fundus. Histopathological evaluation of the mucosal biopsies revealed that the degree of intestinal metaplasia in the upper greater curvature of the gastric body was lower in the NET-positive group than that in the NET-negative group. Although the number of cases in the NET group was limited, this suggests that NETs associated with AIG may begin to develop during the progressive stage of gastritis, even before reaching the terminal phase characterized by fully established intestinal metaplasia. The micronests of gastric endocrine cells, which are considered to be the origin of NETs, are clusters of endocrine cells that proliferate and form nest-like structures outside the glandular ducts within the mucosa. Therefore, gastric NETs may develop independent of the degree of intestinal metaplasia.

In addition to the results of microbiome analysis, metabolome analysis revealed significant changes in the metabolite peak measurements. In patients with AIG, glucose metabolism shifts away from glycolysis toward alternative pathways. This shift, along with a decline in TCA cycle activity, leads to reduced energy production. However, other metabolic pathways may compensate for this decline, suggesting metabolic reprogramming. This alteration is likely to be influenced by mitochondrial dysfunction, an inflammatory environment, enhanced gastric mucosal repair, and epigenetic dysregulation.

Overall, microbiome analysis revealed significant differences between the NET-negative and NET-positive groups, whereas metabolome analysis demonstrated pronounced differences between the control and patients with AIG. In the alluvial diagram generated from the integrated analysis of GMS and tissue metabotypes, the strong association between specific tissue metabotypes and AIG suggests that tissue metabotypes are linked to AIG, while the strong association between specific GMS and the NET-positive group suggests that AIG patients harboring specific GMS may be at increased risk of NET formation. This finding raises the possibility that alterations in host tissue-derived metabolites in patients with AIG may occur prior to shifts in the gastric mucosa-associated microbiota, thereby promoting the formation of a microenvironment permissive for the proliferation of specific microbes implicated in NET pathogenesis.

This study had several limitations. First, the sample size was small, because AIG and associated NETs are relatively rare in Japan. Larger scale, multicenter studies are required to validate these findings. Secondly, the use of 16S rRNA sequencing inherently limits functional and pathway analyses, and the relatively low bacterial biomass, together with metabolites being host-derived rather than bacterial, further complicates the interpretation of mucosal microbiome data when integrating pathway-level findings from host tissue metabolomic analysis. Further research is needed to elucidate the causal mechanisms underlying the interactions among the microbiota, host-derived metabolites, and NETs, and to explore potential clinical applications.

In conclusion, this study highlights the significant alterations in gastric mucosa-associated microbiota profiles in patients with AIG who developed gastric NETs. Microbiome analysis revealed distinct microbial profiles, with mucosal dysbiosis potentially contributing to NET formation. Metabolome analysis has revealed a shift away from glycolysis, reduced TCA cycle activity, and metabolic reprogramming in patients with AIG. Integrated analysis of the microbiome and metabolome suggests that alterations in host metabolism may precede mucosal dysbiosis in patients with AIG and promote the development of a microenvironment facilitating NET formation.

## Supplementary Information

Below is the link to the electronic supplementary material.

**Supplementary material 1. Determination of optimal non-negative matrix factorization rank by 10 repetitions of 3-by-3 bicross validation.** Analysis of operational taxonomic unit abundance data via applications of prevalence filter at (**a**) 0%, (**b**) 1%, (**c**) 4%. (**d**) 8%, (**e**) 12%, (**f**) 16%, (**g**) 20%. OTU, operational taxonomic unit; NMF, non-negative matrix factorization (TIF 6059 KB)

**Supplementary material 2. Determination of appropriate non-negative matrix factorization L1 regularization parameter α by 10 repetitions of 3-by-3 bicross validation.** Analysis of operational taxonomic unit abundance data via applications of prevalence filter at (**a**) 0%, (**b**) 1%, (**c**) 4%. (**d**) 8%, (**e**) 12%, (**f**) 16%, (**g**) 20%. OTU, operational taxonomic unit (TIF 6292 KB)

**Supplementary material 3. Taxonomic relative abundance profiles of gastric microbiota signatures identified by non-negative matrix factorization.** Bar plots represent the arcsine square root-transformed average relative abundance of gastric mucosa-associated microbial taxa. GMS, gastric microbiota signatures (TIF 1539 KB)

**Supplementary material 4** (DOCX 21 KB)

**Supplementary material 5** (DOCX 16 KB)

**Supplementary material 6. Comparative relative abundance analysis of gastric mucosa-associated microbial taxa.** (**a**) Phylum-level composition of gastric mucosa-associated microbiota, (**b**) Genus-level composition of gastric mucosa-associated microbiota, (**c**) Species-level composition of gastric mucosa-associated microbiota (top 50 species after removal of unassigned organisms) (TIF 1165 KB)

## Abbreviations

AIG: Autoimmune gastritis
NETs: Neuroendocrine tumors
ECL: Enterochromaffin-like
*H. pylori*: *Helicobacter pylori*
GI: Gastrointestinal
TE: Tris–EDTA
PCR: Polymerase chain reaction
CE-TOFMS: Capillary electrophoresis time-of-flight mass spectrometry
HMT: Human Metabolome Technologies
*m*/*z*: Mass/charge ratio
MT: Migration time
GMS: Gastric microbiota signatures
FDR: False discovery rate
PERMANOVA: Permutational multivariate analysis of variance
PCoA: Principal coordinate analysis
TCA: Tricarboxylic acid
