# Supplementary figures and images for: Development of gastric mucosa-associated microbiota in autoimmune gastritis with neuroendocrine tumors
Source: J Gastroenterol. 2025 Sep 11;60(12):1481–95. doi: 10.1007/s00535-025-02298-w (PMC12630263; doi:10.1007/s00535-025-02298-w)

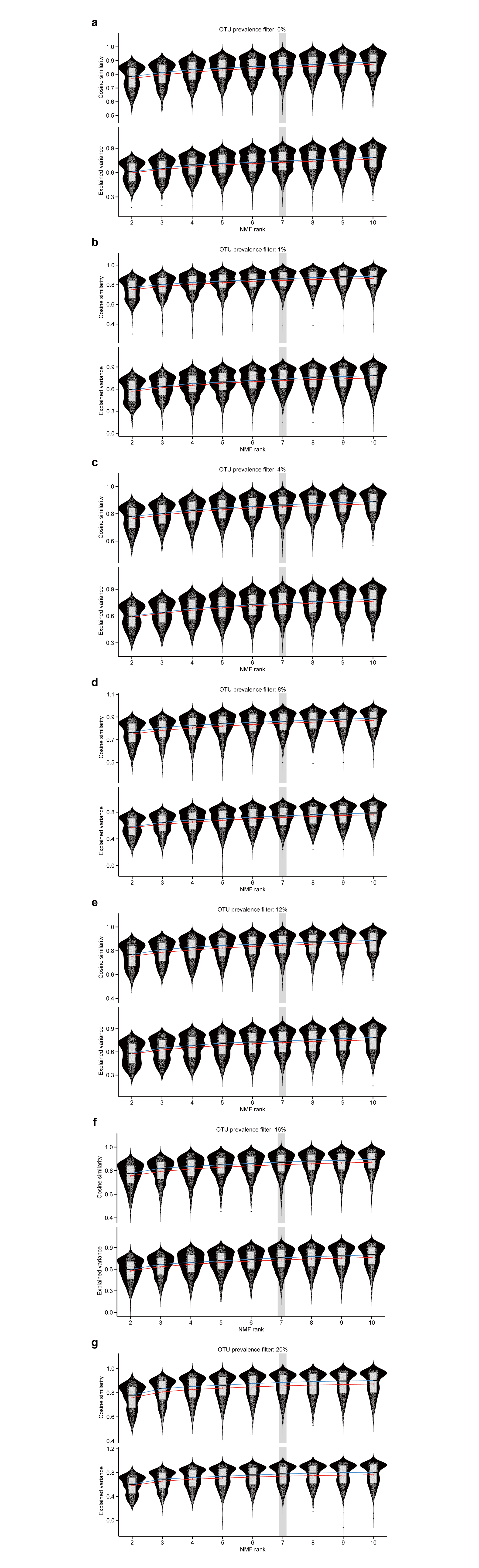

Supplement: Supplementary file 1 — Supplementary material 1. Determination of optimal non-negative matrix factorization rank by 10 repetitions of 3-by-3 bicross validation. Analysis of operational taxonomic unit abundance data via applications of prevalence filter at (a) 0%, (b) 1%, (c) 4%. (d) 8%, (e) 12%, (f) 16%, (g) 20%. OTU, operational taxonomic unit; NMF, non-negative matrix factorization (TIF 6059 KB) [file 535_2025_2298_MOESM1_ESM.tif]

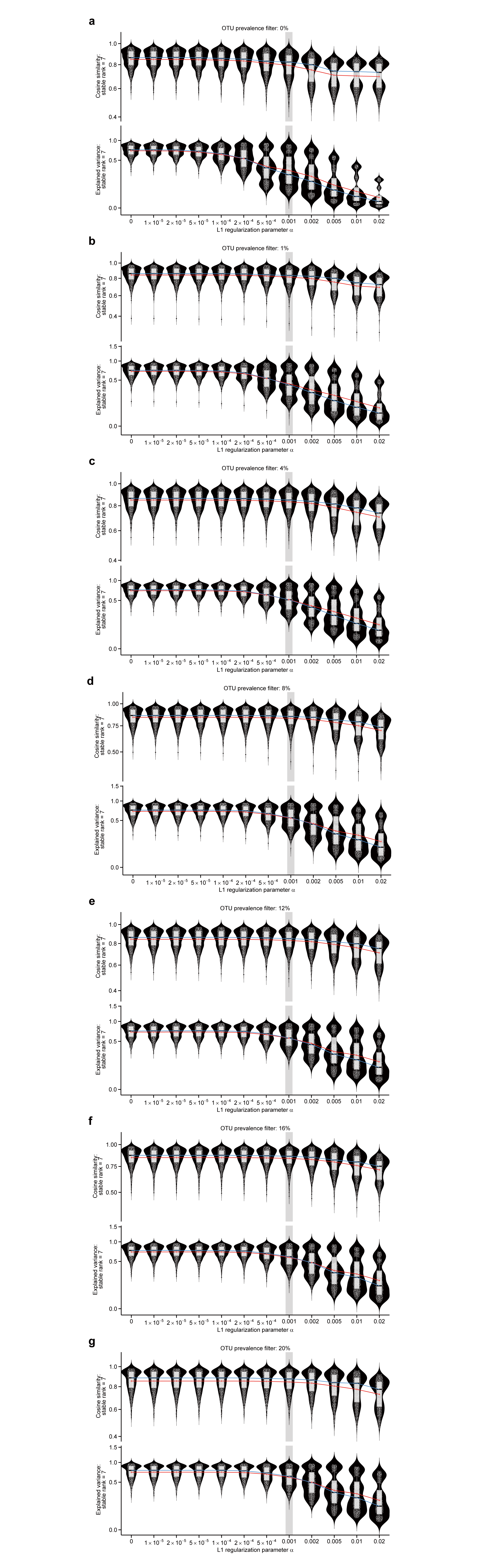

Supplement: Supplementary file 2 — Supplementary material 2. Determination of appropriate non-negative matrix factorization L1 regularization parameter α by 10 repetitions of 3-by-3 bicross validation. Analysis of operational taxonomic unit abundance data via applications of prevalence filter at (a) 0%, (b) 1%, (c) 4%. (d) 8%, (e) 12%, (f) 16%, (g) 20%. OTU, operational taxonomic unit (TIF 6292 KB) [file 535_2025_2298_MOESM2_ESM.tif]

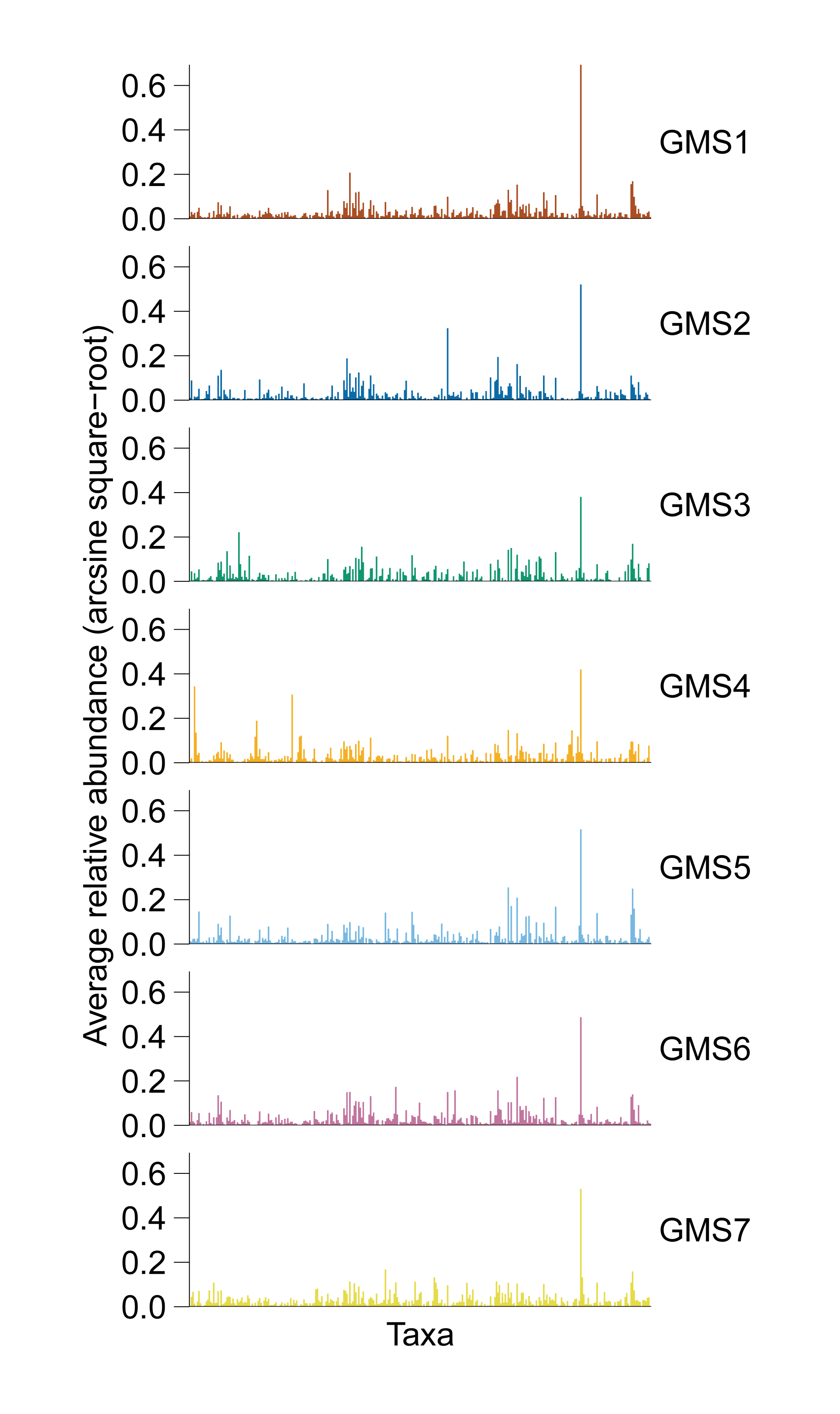

Supplement: Supplementary file 3 — Supplementary material 3. Taxonomic relative abundance profiles of gastric microbiota signatures identified by non-negative matrix factorization. Bar plots represent the arcsine square root-transformed average relative abundance of gastric mucosa-associated microbial taxa. GMS, gastric microbiota signatures (TIF 1539 KB) [file 535_2025_2298_MOESM3_ESM.tif]

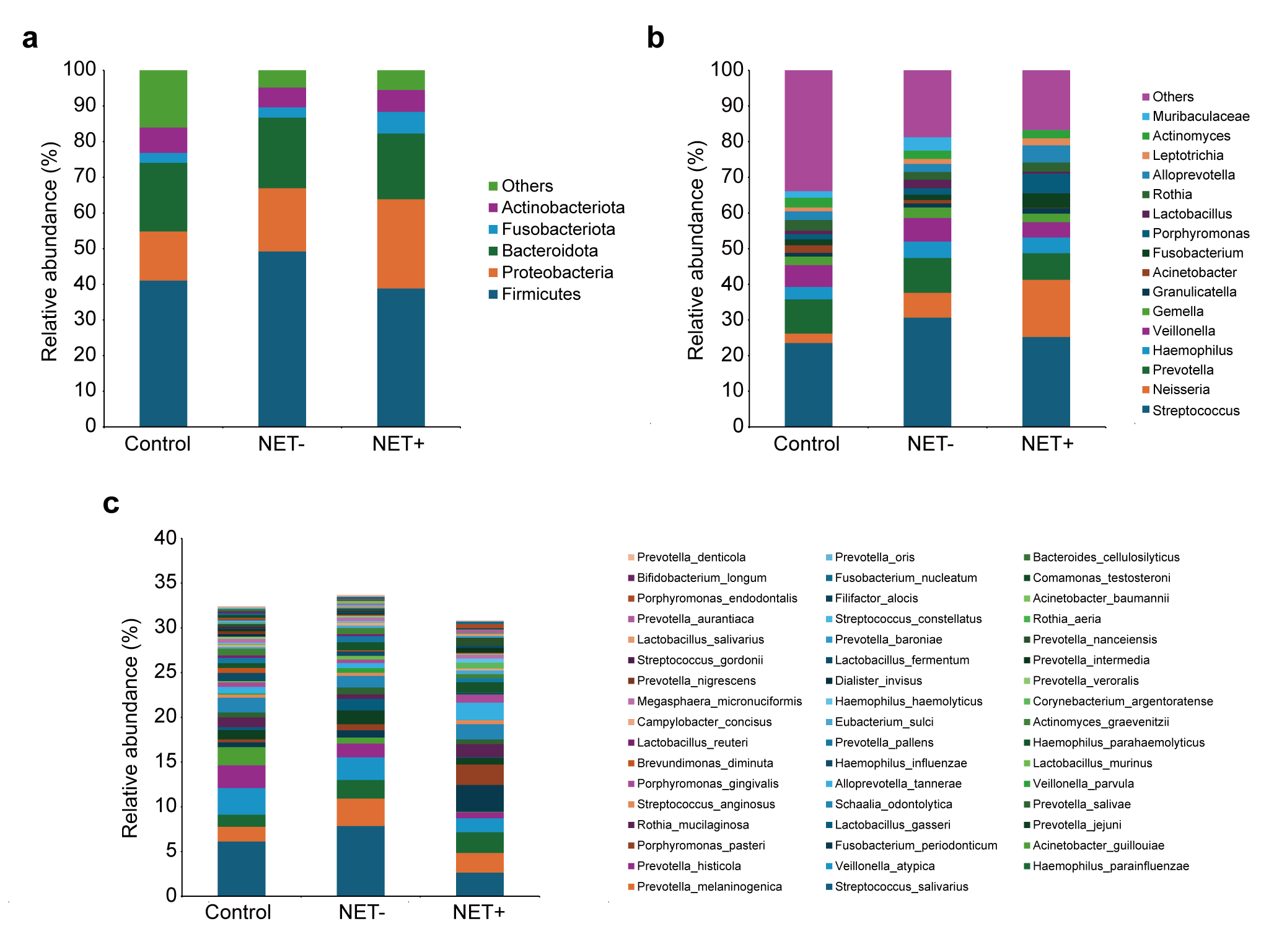

Supplement: Supplementary file 6 — Supplementary material 6. Comparative relative abundance analysis of gastric mucosa-associated microbial taxa. (a) Phylum-level composition of gastric mucosa-associated microbiota, (b) Genus-level composition of gastric mucosa-associated microbiota, (c) Species-level composition of gastric mucosa-associated microbiota (top 50 species after removal of unassigned organisms) (TIF 1165 KB) [file 535_2025_2298_MOESM6_ESM.tif]
