# Supplementary material for: Development of gastric mucosa-associated microbiota in autoimmune gastritis with neuroendocrine tumors
Source: J Gastroenterol. 2025 Sep 11;60(12):1481–95. doi: 10.1007/s00535-025-02298-w (PMC12630263; doi:10.1007/s00535-025-02298-w)
Supplement: Supplementary file 4 — Supplementary material 4 (DOCX 21 KB) [file 535_2025_2298_MOESM4_ESM.docx]

**Supplementary material 4. Upper gastrointestinal endoscopic and histopathological findings of the background gastric mucosa**

| **Variables** | **CL** | **N-** | **N+** | ***p*-value** | | | |
| --- | --- | --- | --- | --- | --- | --- | --- |
|  |  |  |  | **Overall** | **CL vs. N-** | **CL vs. N+** | **N- vs. N+** |
| Number of patients | 12 | 12 | 7 |  |  |  |  |
| Upper GI endoscopic findings |  |  |  |  |  |  |  |
| Reverse atrophy | 0 (0.0%) | 7 (58.3%) | 3 (42.9%) | 0.007 | 0.005 | 0.036 | 0.650 |
| Remnants of oxyntic mucosa | 0 (0.0%) | 6 (50.0%) | 6 (85.7%) | 0.001 | 0.014 | < 0.001 | 0.173 |
| Pseudopolyps | 0 (0.0%) | 7 (58.3%) | 7 (100.0%) | < 0.001 | 0.005 | < 0.001 | 0.106 |
| Sticky adherent dense mucus | 0 (0.0%) | 8 (66.7%) | 3 (42.9%) | 0.003 | 0.001 | 0.036 | 0.377 |
| Scattered minute whitish protrusions | 0 (0.0%) | 1 (8.3%) | 0 (0.0%) | 0.149 | 0.217 | 0.123 | 1.000 |
| Updated Sydney system |  |  |  |  |  |  |  |
| Greater curvature of antrum |  |  |  |  |  |  |  |
| *H. pylori* (-/1+/2+/3+) | 12/0/0/0 | 12/0/0/0 | 7/0/0/0 | NA | NA | NA | NA |
| Neutrophils (-/1+/2+/3+) | 12/0/0/0 | 11/1/0/0 | 7/0/0/0 | 0.441 | 1.000 | 1.000 | 1.000 |
| Mononuclear cells (-/1+/2+/3+) | 6/5/1/0 | 1/10/1/0 | 0/7/0/0 | 0.044 | 0.069 | 0.025 | 1.000 |
| Atrophy (-/1+/2+/3+) | 12/0/0/0 | 7/5/0/0 | 3/4/0/0 | 0.014 | 0.037 | 0.009 | 0.650 |
| Intestinal metaplasia (-/1+/2+/3+) | 12/0/0/0 | 10/2/0/0 | 7/0/0/0 | 0.184 | 0.478 | 1.000 | 0.509 |
| Lesser curvature of angulus |  |  |  |  |  |  |  |
| *H. pylori* (-/1+/2+/3+) | 12/0/0/0 | 12/0/0/0 | 7/0/0/0 | NA | NA | NA | NA |
| Neutrophils (-/1+/2+/3+) | 12/0/0/0 | 11/1/0/0 | 7/0/0/0 | 0.441 | 1.000 | 1.000 | 1.000 |
| Mononuclear cells (-/1+/2+/3+) | 5/5/2/0 | 1/10/1/0 | 0/7/0/0 | 0.060 | 0.122 | 0.061 | 1.000 |
| Atrophy (-/1+/2+/3+) | 12/0/0/0 | 4/7/1/0 | 4/3/0/0 | 0.014 | 0.001 | 0.036 | 0.767 |
| Intestinal metaplasia (-/1+/2+/3+) | 12/0/0/0 | 9/3/0/0 | 7/0/0/0 | 0.072 | 0.217 | 1.000 | 0.263 |
| Lesser curvature of corpus |  |  |  |  |  |  |  |
| *H. pylori* (-/1+/2+/3+) | 12/0/0/0 | 12/0/0/0 | 7/0/0/0 | NA | NA | NA | NA |
| Neutrophils (-/1+/2+/3+) | 12/0/0/0 | 11/1/0/0 | 7/0/0/0 | 0.441 | 1.000 | 1.000 | 1.000 |
| Mononuclear cells (-/1+/2+/3+) | 4/6/2/0 | 0/9/3/0 | 0/7/0/0 | 0.047 | 0.125 | 0.109 | 0.263 |
| Atrophy (-/1+/2+/3+) | 12/0/0/0 | 1/7/4/0 | 2/5/0/0 | < 0.001 | < 0.001 | 0.002 | 0.163 |
| Intestinal metaplasia (-/1+/2+/3+) | 12/0/0/0 | 2/7/3/0 | 5/2/0/0 | 0.001 | < 0.001 | 0.123 | 0.090 |
| Greater curvature of corpus |  |  |  |  |  |  |  |
| *H. pylori* (-/1+/2+/3+) | 12/0/0/0 | 12/0/0/0 | 7/0/0/0 | NA | NA | NA | NA |
| Neutrophils (-/1+/2+/3+) | 12/0/0/0 | 12/0/0/0 | 7/0/0/0 | NA | NA | NA | NA |
| Mononuclear cells (-/1+/2+/3+) | 4/6/2/0 | 0/9/3/0 | 0/6/1/0 | 0.105 | 0.125 | 0.270 | 1.000 |
| Atrophy (-/1+/2+/3+) | 12/0/0/0 | 1/8/2/1 | 2/5/0/0 | < 0.001 | < 0.001 | 0.002 | 0.574 |
| Intestinal metaplasia (-/1+/2+/3+) | 12/0/0/0 | 1/9/2/0 | 5/2/0/0 | < 0.001 | < 0.001 | 0.123 | 0.019 |
| ECL cell hyperplasia | 0 (0.0%) | 6 (50.0%) | 5 (71.4%) | 0.003 | 0.014 | 0.002 | 0.633 |

CL, control group; N−, neuroendocrine-negative group; N+, neuroendocrine-positive group; GI, gastrointestinal; *H. pylori*, *Helicobacter pylori*; ECL, enterochromaffin-like; NA, not available
