# Supplementary material for: Development of gastric mucosa-associated microbiota in autoimmune gastritis with neuroendocrine tumors
Source: J Gastroenterol. 2025 Sep 11;60(12):1481–95. doi: 10.1007/s00535-025-02298-w (PMC12630263; doi:10.1007/s00535-025-02298-w)
Supplement: Supplementary file 5 — Supplementary material 5 (DOCX 16 KB) [file 535_2025_2298_MOESM5_ESM.docx]

**Supplementary material 5. Characteristics of neuroendocrine tumor lesions occurring in patients with autoimmune gastritis**

| **Variables** | **N+** |
| --- | --- |
| Number of patients | 7 |
| Number of lesions | 12 |
| Size (mm) (Median [IQR]) | 5.5 (4.5, 6.0) |
| Location |  |
| Greater curvature of angulus | 1 (8.3%) |
| Anterior wall of lower corpus | 1 (8.3%) |
| Lesser curvature of lower corpus | 1 (8.3%) |
| Greater curvature of lower corpus | 2 (16.7%) |
| Lesser curvature of middle corpus | 1 (8.3%) |
| Anterior wall of upper corpus | 1 (8.3%) |
| Lesser curvature of upper corpus | 1 (8.3%) |
| Greater curvature of upper corpus | 3 (25.0%) |
| Fundus | 1 (8.3%) |
| Treatment |  |
| ESD | 9 (75.0%) |
| Disappeared after biopsy | 2 (16.7%) |
| Observation after biopsy | 1 (8.3%) |
| WHO classification |  |
| NETG1 | 12 (100.0%) |

N+, neuroendocrine-positive group; IQR, interquartile range; ESD, endoscopic submucosal dissection, WHO, world health organization, NETG1, neuroendocrine tumor grade 1
